# Harnessing barley grains for green synthesis of gold and silver nanoparticles with antibacterial potential

**DOI:** 10.1186/s11671-024-04042-4

**Published:** 2024-06-11

**Authors:** Priyanka Singh, Ivan Mijakovic

**Affiliations:** 1 The Novo Nordisk Foundation, Center for Biosustainability, Technical University of Denmark, 2800 Kongens Lyngby, Denmark; 2 Systems and Synthetic Biology Division, Department of Biology and Biological Engineering, Chalmers University of Technology, 412 96 Gothenburg, Sweden

**Keywords:** Gold nanoparticles, Silver nanoparticles, Barley, Stability, Antibacterial activity

## Abstract

The continuous evolution and significance of green resources-based nanomaterials have spurred the exploration of sustainable sources for nanoparticle production. Green synthesis routes offer eco-friendly methodologies, ensuring nanoparticle stability and monodispersity, enhancing their efficiency for various applications. Notably, the thick biological corona layer surrounding nanoparticles (NPs) synthesized through green routes contributes to their unique properties. Consequently, there has been a surge in the development of NPs synthesis methods utilizing medicinal plants and diverse agricultural and waste resources. This study highlights the sustainable potential of barley grains for the synthesis of gold nanoparticles (Barley-AuNPs) and silver nanoparticles (Barley-AgNPs) as an environmentally friendly alternative, followed by NPs characterizations and their application against pathogenic bacteria: *Escherichia coli* UTI 89 and *Pseudomonas aeruginosa* PAO1. The rapid synthesis of Barley-AuNPs within 20 min and Barley-AgNPs within 30 min at 90 °C underscores the efficiency of barley as a green precursor. Characterization through advanced techniques, including SEM, TEM, EDS, AFM, DLS, FT-IR, MALDI-TOF, and sp-ICPMS, reveals the 20–25 nm size for Barley-AuNPs, while Barley-AgNPs demonstrate 2–10 nm size with spherical monodispersity. A notable contribution lies in the stability of these NPs over extended periods, attributed to a thick biological corona layer. This corona layer, which enhances stability, also influences the antimicrobial activity of Barley-AgNPs, presenting an intriguing trade-off. The antimicrobial investigations highlight the significant potential of Barley-AgNPs, with distinct minimum bactericidal concentrations (MBC) against *P. aeruginosa* and *E. coli* at 8 µg/mL. Overall, this research pioneers the use of barley grains for nanoparticle synthesis and unveils these nanoparticles' unique characteristics and potential antibacterial applications, contributing to the evolving landscape of sustainable nanotechnology.

**Graphic Abstract:**

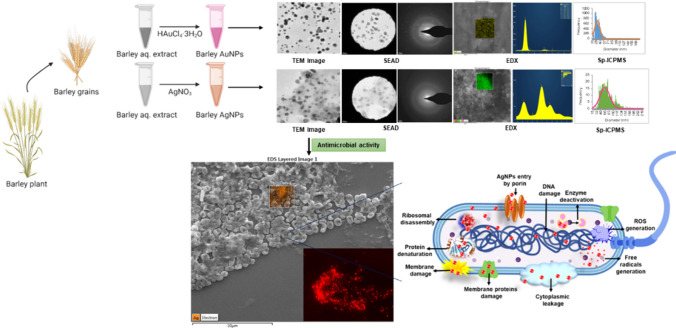

## Introduction

In the relentless pursuit of generating biocompatible nanoparticles, exploring innovative technologies and resources has become paramount. Various physio-chemical methods have been reported, which lead to the quick generation of nanoparticles. Still, most are expensive, resulting in hazardous byproducts and waste generation [1]. One effective avenue is utilizing biological sources, such as microorganisms and plants, for nanoparticle synthesis, providing an eco-friendly alternative to traditional methods [2]. While microbial synthesis has demonstrated efficiency, it has drawbacks, including the need for specific growth conditions, energy-intensive incubators, and complex purification processes [3, 4]. To overcome these limitations, the focus has shifted towards green synthesis using plants [5], which offers a rapid, facile, and energy-efficient method for nanoparticle production [6]. Unlike microbial synthesis, plant-mediated nanoparticle production occurs within seconds to minutes, resulting in stable, biocompatible, and monodisperse NPs [7–9]. Medicinal plants, in particular, have garnered attention for their ability to imbue NPs with medicinal components, enhancing their efficacy for various applications [10]. Furthermore, exploring waste products from plants, such as onion peels or plants' fallen leaves, fruits, and barks for nanoparticle synthesis, highlights the potential for generating valuable products while repurposing agricultural by-products [11–13]. In this context, the study delves into synthesizing gold and silver NPs using barley grains. Barley grains, renowned for their versatility and widespread cultivation, present a unique opportunity for nanoparticle synthesis owing to their numerous benefits and sustainable attributes. Barley grains are readily available on a large scale, making them a cost-effective resource for nanoparticle production. Their abundance ensures a consistent and reliable supply, reducing dependency on fluctuating market conditions or seasonal variations. Moreover, the cultivation of barley is relatively sustainable, requiring minimal inputs such as water and fertilizers compared to other crops, thus aligning with eco-friendly practices.

The physiological properties of NPs, notably their shape and size, are fundamental determinants significantly influencing their behavior and application potential [14]. Their distinctive characteristics contribute to various properties, including optical, electronic, and catalytic functionalities, making them highly adaptable across multiple domains. These NPs possess unique attributes that render them indispensable tools for advancing medical technologies and tackling various healthcare challenges. Our study centered on the synthesis and application potential of gold and silver NPs, recognizing their versatility and significant promise, particularly within the biomedical field. AuNPs exhibit diverse diagnostics, sensor development, photo imaging, and photothermal therapy applications [15, 16]. Their unique optical properties, finely tunable through size and shape control, underscore their versatility and excellent biocompatibility, positioning them as pivotal components in various medical technologies. Notably, AuNPs play a pivotal role in targeted drug delivery systems and cancer treatment, capitalizing on their selective accumulation in specific tissues and cells [17, 18]. Conversely, AgNPs demonstrate remarkable antimicrobial properties against numerous multi-drug-resistant pathogens, extending their relevance beyond biomedicine [19, 20]. They are widely employed in applications such as antimicrobial, anticancer, and anti-inflammatory agents, with their efficacy linked to size and shape-dependent properties [21–23]. This versatility has led to their utilization in textiles, packaging, medical devices, and cosmetics, addressing the critical need for effective infection control in healthcare settings [10, 21]. The tailored manipulation of NPs properties is imperative for optimizing their performance in specific applications as nanotechnology advances, facilitating innovative developments in diagnostics, therapeutics, and materials science [24]. Here, in particular, we tried to explore the barley-AgNPs against human pathogens *P. aeruginosa* and *E. coli.*

Thus, in this study, we harness the potential of barley plants for the green synthesis of gold and silver NPs. By utilizing barley, an abundant and often overlooked resource, we seek to produce highly demanded NPs sustainably. Through comprehensive analytical characterizations, we aim to elucidate the unique properties of the synthesized NPs. Furthermore, the study investigates the antimicrobial potential of Barley-AgNPs against Gram-negative pathogens, specifically *P. aeruginosa* and *E. coli*. This research contributes to the evolving landscape of green nanotechnology, emphasizing the importance of sustainable resources in nanoparticle synthesis and their potential applications in diverse fields.

## Materials and methods

### Materials

We obtained high-purity gold(III) chloride trihydrate (HAuCl_4_·3H_2_O) and silver nitrate (AgNO_3_) from Sigma–Aldrich Chemicals (St Louis, MO, USA). Dried barley grains were carefully cleaned by washing them twice with distilled water to remove impurities. After air-drying overnight, 10 g of grains were mixed with 90 mL of distilled water in a sterile flask and autoclaved at 100 °C for 20 min [3, 4]. Following autoclaving, the mixture underwent filtration to remove any remaining particulates, and additional purification was achieved through centrifugation at 8000 rpm for 3 min to eliminate fine suspended residues. The resulting liquid, considered a stock solution, was then used for nanoparticle production in various dilutions. The aqueous extract was further adjusted by diluting it with different distilled water ratios, forming what we refer to as a synthesis medium (SM) [3].

### Green synthesis of Barley-AuNPs and Barley-AgNPs

In synthesizing NPs, we employed an concentration of 1 mM of HAuCl_4_·3H_2_O and AgNO_3_, carefully added to an SM separately [13]. The SM underwent incubation at specific time and temperature conditions to facilitate nanoparticle formation. The initiation of nanoparticle synthesis was initially gauged by observing color changes in the SM, followed by spectral analysis. Upon successful nanoparticle formation, optimization studies for salt concentration (AuNPs 0.5–2.5 mM) and (AgNPs 1–12 mM), temperature 60–90 °C, and 1–30 min were explored for both the NP types. Purification was undertaken through a two-step centrifugation process. Initially, centrifugation at 2000 rpm for 5 min facilitated the removal of larger particulates, followed by subsequent centrifugation at 14,000 rpm for 10 min to collect the fine NPs [25]. Furthermore, the NPs underwent a triple wash with distilled water to eliminate residual metal ions or other constituents. The finalized NPs were then gathered into a pellet and suspended in distilled water, serving as the medium for subsequent analytical characterization and application studies [26, 27]. For detailed analyses such as thermogravimetric analysis (TGA) and Fourier Transform-Infrared Spectroscopy (FT-IR), the NPs were air-dried to form a pellet, ensuring optimal conditions for accurate characterization [3].

### Analytical characterization of Barley-AuNPs and Barley-AgNPs

The analytical characterization of Barley-AuNPs and Barley-AgNPs involved various advanced techniques and instruments to unravel their structural, morphological, and chemical properties. Visible inspection and UV–Vis spectroscopy were employed to monitor the reduction of gold and silver ions using the JENWAY 6705 UV–Vis spectrophotometer. This instrument facilitated the scanning of the SM within the 300–700 nm range, with visible and UV–Vis spectrum analyses pivotal for optimizing the production of Barley-AuNPs and Barley-AgNPs [26].

To quantify the concentration of the NPs, single-particle inductively coupled plasma-mass spectrometry (sp-ICP-MS) was utilized. The NexION 350D by PerkinElmer Inc. offered exceptional sensitivity for measuring individual NPs, which is crucial in stability assessments under varying conditions [4]. Thermogravimetric Analysis (TGA), conducted using the TA Instruments instrument, allowed for an evaluation of the temperature stability of the NPs. Dried pellets of NPs underwent temperature ramping from 20 to 700 °C at 10 °C/min. Scanning Electron Microscopy with Energy Dispersive X-ray (SEM–EDX), coupled with the Quanta FEG 200 ESEM microscope, provided insights into the morphology and elemental composition of Barley-AuNPs and Barley-AgNPs. This high-resolution imaging technique facilitated elemental analysis through EDX. Transmission Electron Microscopy (TEM) using FEI Tecnai T20 G2 delivered detailed structural morphology and crystallographic information. Operating at an acceleration voltage of 200 kV, this instrument enabled a close examination of Barley-AuNPs and Barley-AgNPs. Atomic force microscopy (AFM) investigations were conducted employing intermittent contact mode on a Park NX20 instrument obtained from www.parkafm.com. The utilized probes were standard single-crystal highly doped silicon probes featuring a radius of curvature below 30 nm (SuperSharpSiliconTM Non-Contact AFM probes sourced from Nanosensors). The standard uncertainty (u(d)) associated with the determined diameters is constrained to be less than 5% of the measured values, expressed as u(d) < 0.05 d [26].

Dynamic Light Scattering (DLS) measurements were performed using the Zetasizer Nano ZS, Chuo-ku Kobe-shi, Japan, offering valuable information about the size distribution and zeta potential of the NPs. Autocorrelation functions were analyzed using the Contin algorithm. FT-IR analysis, conducted with the Nicolet iS50 by ThermoFisher Scientific, identified biomolecules and functional groups responsible for the reduction, capping, and stabilization of Barley-AuNPs and Barley-AgNPs. The instrument scanned the purified pellet of NPs and freeze-dried barley grains aqueous extract. MALDI-TOF Mass Spectrometry, performed with the Ultraflex II by Bruker-Daltonics, involved loading purified NPs onto an AnchorChipTM target plate. This technique provided detailed mass distribution information for Barley-AuNPs and Barley-AgNPs, with spectra processed and calibrated using Flex Analysis v3.0 and protein standards [28].

### Antibacterial activity of Barley-AgNPs against human pathogens

In investigating the antibacterial activity of Barley-AgNPs, the focus was directed towards their effects on Gram-negative pathogens, specifically *Escherichia coli* UTI 89 and *Pseudomonas aeruginosa* PAO1. The cultures of these bacterial strains were grown overnight in LB medium at 37 °C. Then the optical density (OD550) of the cultures was adjusted to 1–2 × 10^5^ colony forming units (CFU)/mL by appropriate dilution. Barley-AgNPs were introduced at concentrations ranging from 1 to 8 µg/mL, and the mixture was incubated for 24 h at 37 °C with constant shaking. Optical density (OD) at 550 nm was measured after the incubation period to assess the impact of Barley-AgNPs. The Minimum Bactericidal Concentration (MBC) value, indicative of the lowest concentration of Barley-AgNPs required for bacterial elimination, was determined by spreading 100 µL of the culture on agar plates and subsequent CFU counting. Live/Dead staining was employed using the Live/Dead BacLight Viability kit to visualize the viability of bacterial cells. Both control and treated cells underwent staining with a mixture of 6.0 μM SYTO 9 and 30 μM KI for 20 min. Fluorescence microscopic imaging using a LEICA DM 4000 B revealed the differential staining pattern, distinguishing between live and dead cells. Furthermore, scanning electron microscopy (SEM) analysis was conducted to delve into the structural effects of Barley-AgNPs on individual bacterial cells after fixing the cells with 3% glutaraldehyde overnight at 4 °C, a dehydration process involving graded ethanol concentrations (40, 50, 60, 70, 80, and 90%) for 15 min and with absolute ethanol for 20 min as carried out. The dehydrated samples were placed on SEM carbon tape, air-dried, and coated with gold before imaging. In addition to SEM imaging, EDX analysis and elemental mapping were performed to confirm that the observed antibacterial effects were attributed to the action of Barley-AgNPs [13].

## Results

**Fig. 1:**
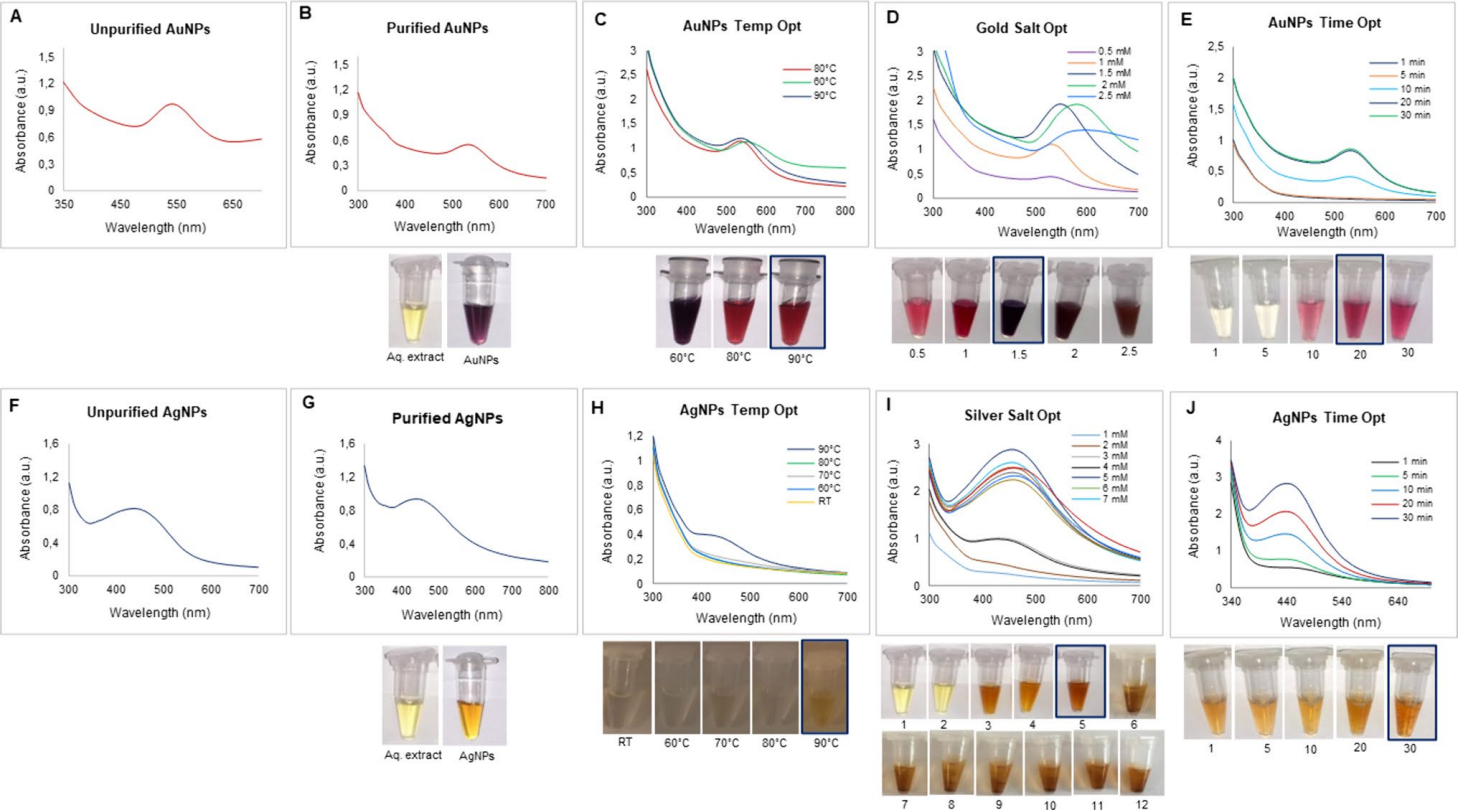
Optimization studies for Barley-AuNPs and Barley-AgNPs production. For Barley-AuNPs: **A** UV–Vis spectrum of unpurified NPs, **B** visible picture and UV–Vis spectrum of purified NPs, **C** temperature optimization, **D** gold salt concentration optimization; **E** synthesis time optimization. For Barley-AgNPs: **F** UV–Vis spectrum of unpurified NPs, **G** visible picture and UV–Vis spectrum of purified NPs, **H** temperature optimization, **I** silver salt concentration optimization; **J** synthesis time optimization

The synthesis of Barley-AuNPs and Barley-AgNPs was a dynamic process, meticulously observed through visible changes and UV–Vis spectroscopy. With its inherent reducing properties, Barley extract effectively facilitated the reduction of gold and silver salts, forming Barley-AuNPs and Barley-AgNPs, respectively. A distinct color change in the SM visually marked the reduction process. Barley-AuNPs presented as a dark purple color (Fig. 1A, B), while Barley-AgNPs displayed a brown color (Fig. 1F, G), in stark contrast to the light yellow color of the barley extract. This visual transformation was indicative of the surface plasmon resonance (SPR) property of the formed NPs. Subsequent UV–Vis spectrum analysis within 300 to 700 nm confirmed the synthesis success. Barley-AuNPs exhibited a clear peak in the 500–600 nm region, while Barley-AgNPs showcased a prominent and broad peak in the 400–500 nm region [20]. The stability of the NPs in solution was affirmed through purified samples, subjected to three cycles of centrifugation after DI water washing, retaining a high-intensity peak in the UV–Vis spectrum [29].

Optimization studies were crucial in tailoring the synthesis conditions for Barley-AuNPs and Barley-AgNPs. Careful consideration of the gold and silver salt concentrations, temperature, salt concentration, and other reaction parameters allowed the identification of optimal conditions. For Barley-AuNPs, the optimal conditions were determined as a reaction temperature of 90 °C, 1.5 mM gold salt concentration, and a reaction time of 20 min at room temperature (Fig. 1C-E). In contrast, Barley-AgNPs optimization included a reaction temperature of 90 °C, 5 mM silver salt concentration, and a synthesis time of 30 min (Fig. 1H-J).

**Fig. 2:**
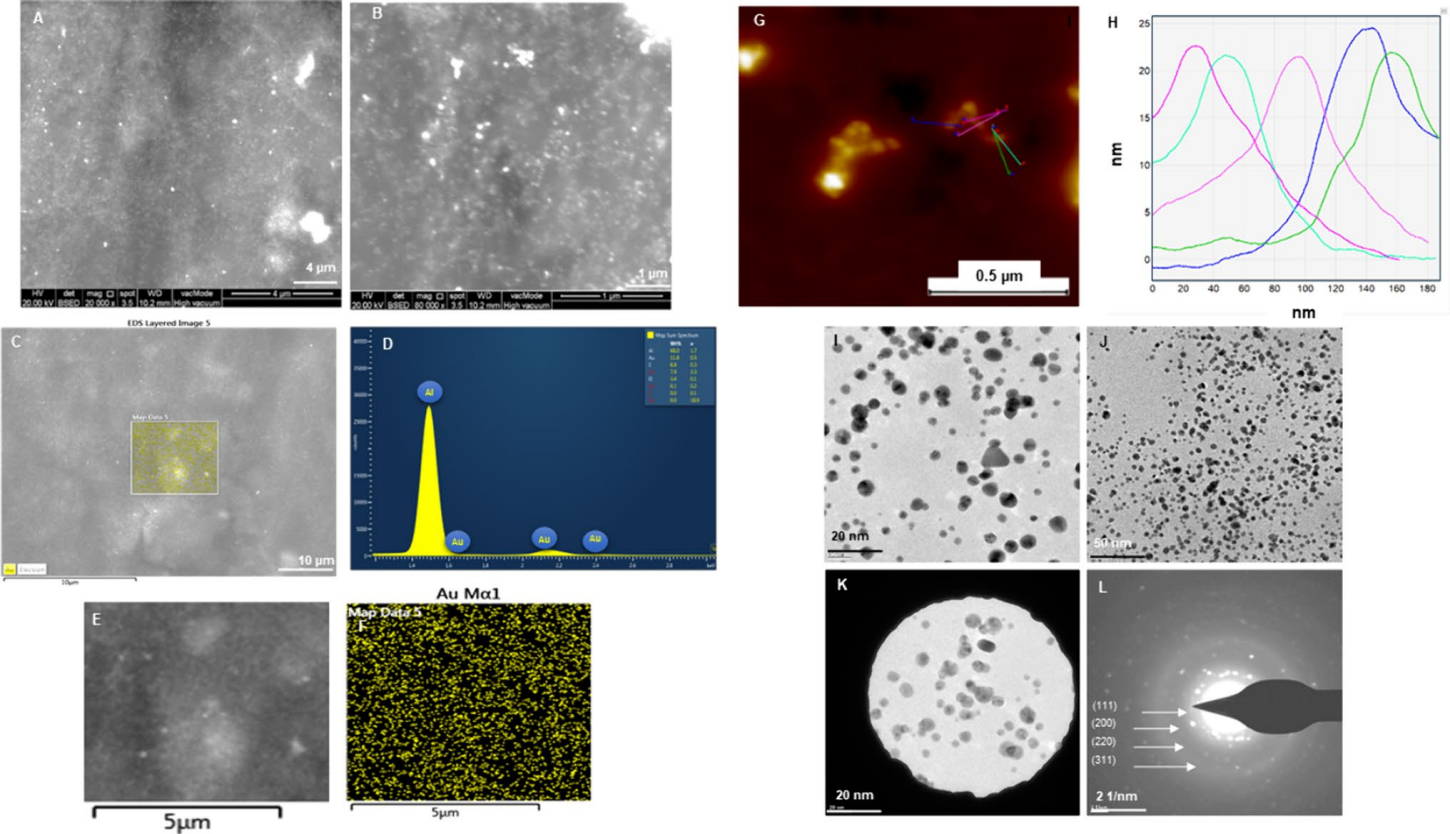
Structural analysis of Barley-AuNPs. **A**, **B** SEM images of NPs at different scales; **C**–**F** Elemental mapping showing scanned image of NPs with gold element distribution (yellow color) and respective EDX spectrum of the elemental mapped region showing sharp peak for gold element; **G**, **H** AFM analysis of NPs showing size distribution; **I**, **J** TEM images at different scales; **K**, **L** SAED pattern of NPs

Characterization of Barley-AuNPs and Barley-AgNPs involved comprehensively exploring their morphological features through SEM, EDX, elemental mapping, TEM, and SAED [20]. SEM and TEM images revealed a diverse population of spherical shape Barley-AuNPs, contributing to monodispersity (Fig. 2). TEM analysis provided insight into the shapes and the presence of an organic layer derived from the extract covering the NPs. EDX and elemental mapping of SEM-scanned images confirmed the distribution of gold elements in Barley-AuNPs [12]. Additionally, TEM images demonstrated the spherical shape of Barley-AuNPs with a core diameter of 20–30 nm with few polydispersities. AFM analysis also appears to be in a similar line with 20–25 nm height.

**Fig. 3:**
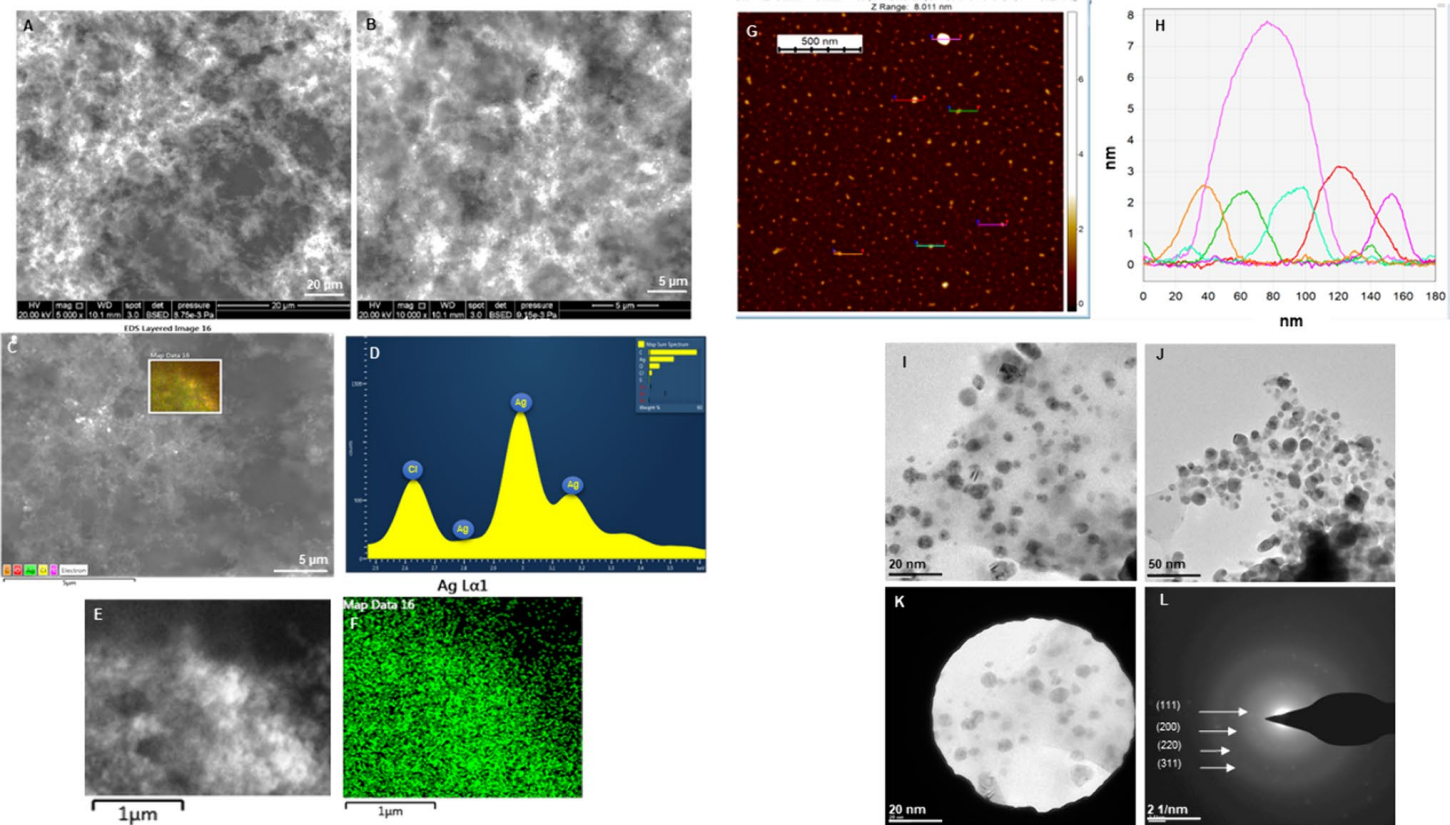
Structural analysis of Barley-AgNPs. **A**, **B** SEM images of NPs at different scales, **C**–**F** Elemental mapping showing scanned image of NPs with silver element distribution (green color), and respective EDX spectrum of the elemental mapped region showing sharp peak for silver element. **G**, **H** AFM analysis of NPs showing size distribution, **I**, **J** TEM images at different scale, **K**, **L** SAED pattern of NPs

Similarly, Barley-AgNPs SEM and TEM results predominantly exhibited spherical morphology with a size of 2–20 nm (Fig. 3) [30]. The SAED patterns indicated the polycrystalline nature of both the NP types [31]. Furthermore, the AFM investigation unveiled a consistent size distribution within the 2–10 nm Barley-AgNPs range. This observation suggests a homogeneity in the dimensions of the analyzed entities [3].

**Fig. 4:**
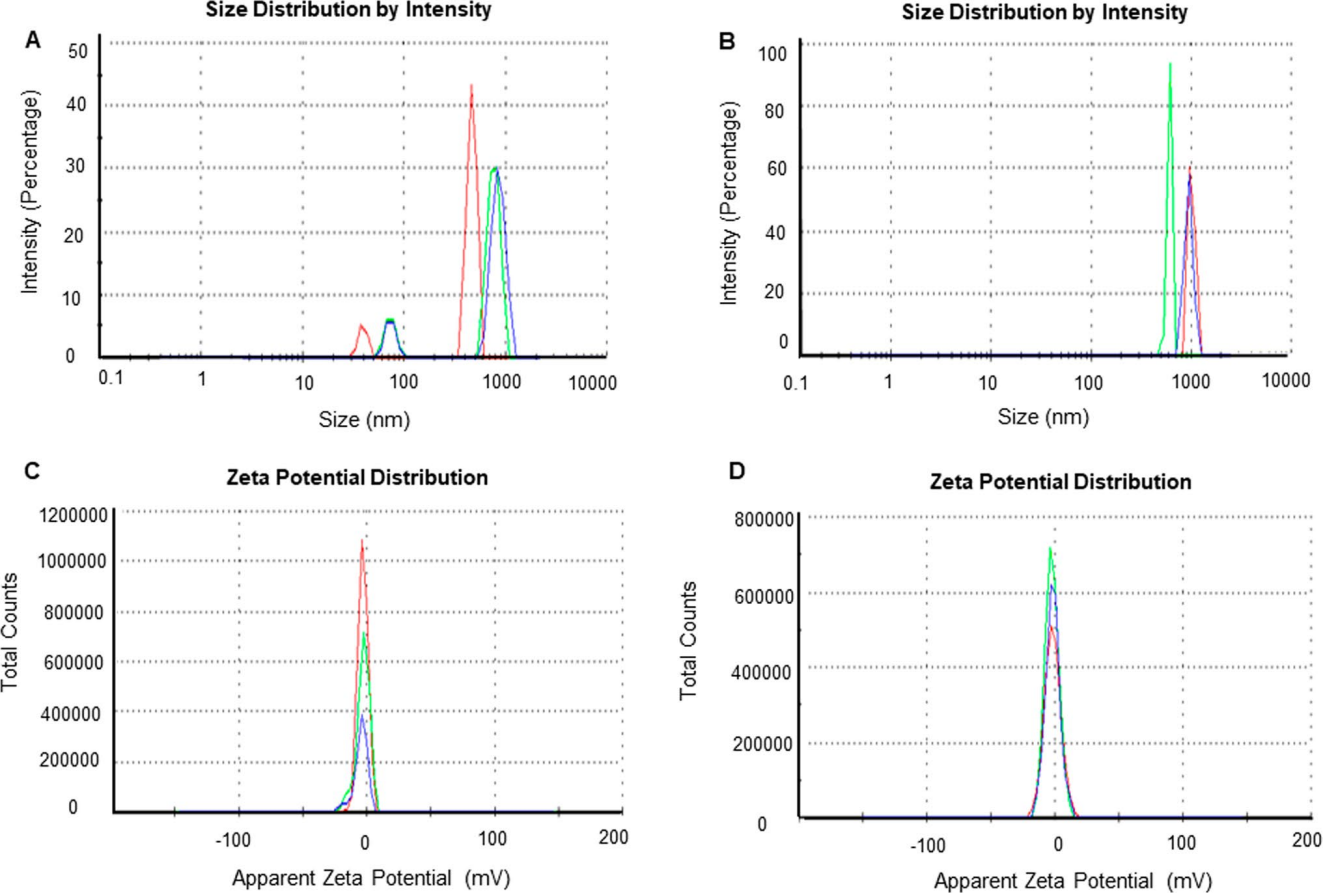
DLS analysis of Barley-AuNPs and Barley-AgNPs. **A** Barley-AuNPs distribution concerning size and intensity. **B** Barley-AgNPs distribution concerning size and intensity. **C** Zeta potential of Barley-AuNPs and **D** Barley-AgNPs, representing highly negative surface charge

**Fig. 5:**
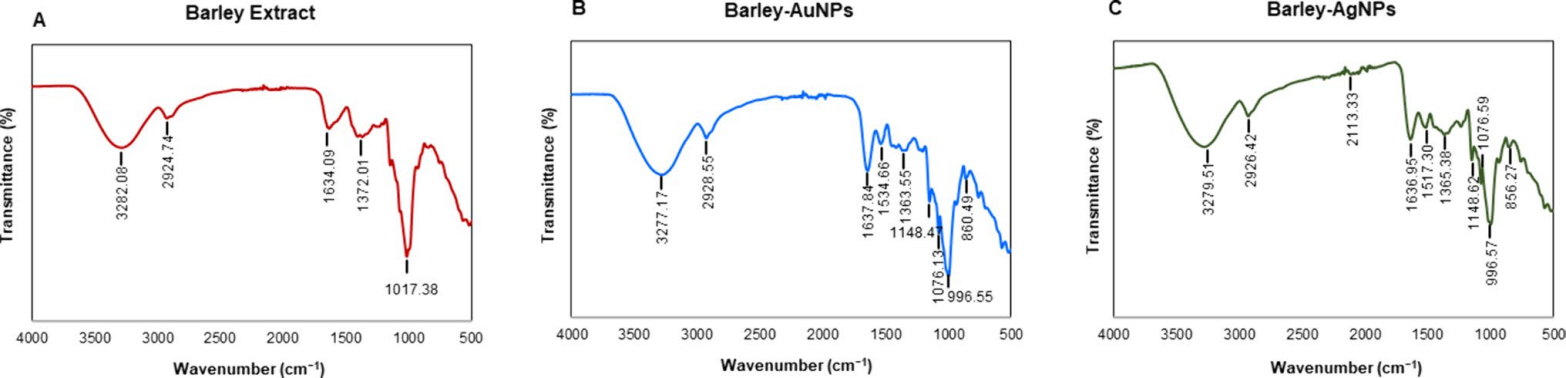
FTIR spectrum of **A** freeze-dried aqueous extract of barley powder and **B** Barley-AuNPs, and **C** Barley-AgNPs, which demonstrate the active surface groups for respective samples

**Fig. 6:**
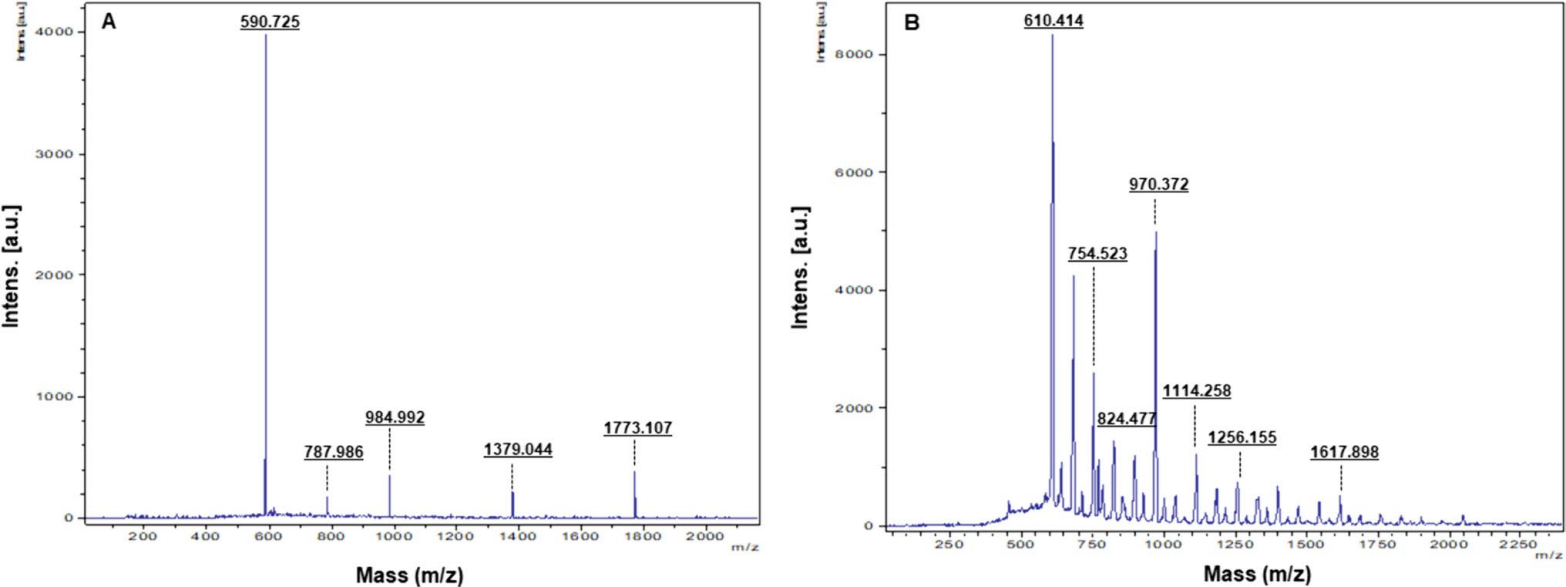
MALDI-TOF analysis of **A** Barley-AuNPs and **B** Barley-AgNPs demonstrate respective ions

**Fig. 7:**
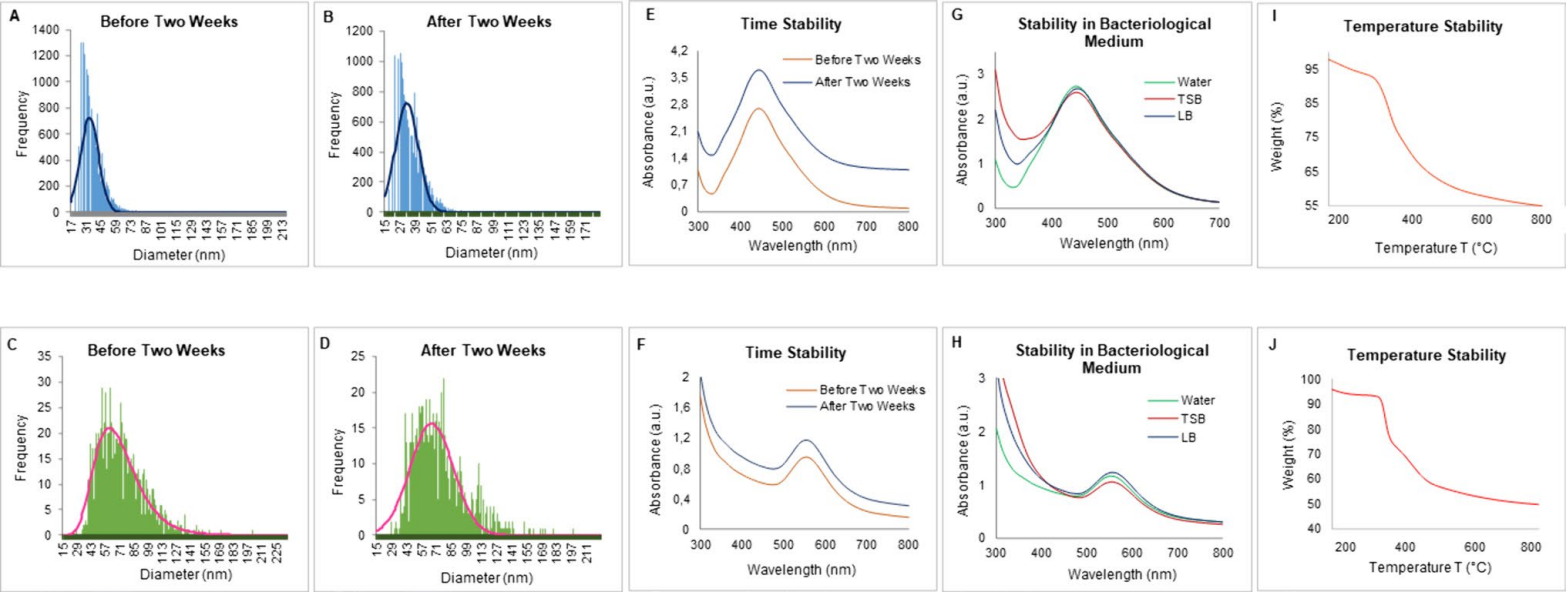
sp-ICPMS and stability analysis of Barley-AuNPs and Barley-AgNPs. ICPMS histogram of Barley-AuNPs at different time intervals **A** fresh Barley-AuNPs, **B** after two weeks. ICPMS histogram of Barley-AgNPs at different time intervals, **C** fresh Barley-AgNPs, **D** after two weeks. UV–Vis spectrum representing the stability analysis of Barley-AuNPs and Barley-AgNPs. Barley-AuNPs: **E** before and after two weeks of incubation at RT, **G** in a different medium, and **I** at the temperature range from 20 to 700 °C measured by the TGA instrument. Barley-AgNPs: **F** before and after two weeks of incubation at RT for, **H** in a different medium, **J** at the temperature range from 20 to 700 °C measured by TGA instrument

DLS analysis provided information on the hydrodynamic diameter and zeta potential values. Barley-AuNPs exhibited a size of 1565 nm with a polydispersity index (PDI) of 0.9 (Fig. 4A), while Barley-AgNPs (Fig. 4B) displayed a size of 4989 nm with a PDI of 0.7 [32]. The zeta potential values indicated highly negative surface charges for both Barley-AuNPs (− 4.73 mV) (Fig. 4C) and Barley-AgNPs (− 1.41 mV) (Fig. 4D), highlighting their negative surface charge. The DLS size is extremely different from TEM and AFM size information, which is attributed to the hydrodynamic diameter of NPs. This information also highlights the presence of a thick corona layer around the NPs, which makes 20–30 nm AuNPs to 1565 nm and 2–20 nm AgNPs to 4989 nm. The AgNPs resemble thicker and more complex surrounding layers than those AuNPs formed here. FT-IR analysis was employed to identify possible functional groups on the NPs' surface [33]. The FT-IR spectrum of Barley extract revealed the presence of various active groups, including hydroxyl groups, amine N–H/O–H stretching vibrations, methyl groups, flavonoids, terpenoids, proteins, and amino acids. The FT-IR spectra of Barley-AuNPs and Barley-AgNPs exhibited overlapping peaks in similar regions, indicating the presence of multiple active surface groups derived from the barley extract (Table 1) (Fig. 5A–C). MALDI-TOF analysis provided insights into the protein content on the surface of Barley-AuNPs and Barley-AgNPs (Fig. 6A, B). Mass spectra showed intense peaks assigned to gold and silver ions, further confirming the proteinaceous nature of the NPs [28]. sp-ICPMS analysis provided insights into the concentration, yield, and stability of Barley-AuNPs (Fig. 7A, B) and Barley-AgNPs (Fig. 7C, D) over different time intervals. Concentrations were measured as 0.66 mg/L for Barley-AuNPs and 0.04 mg/L for Barley-AgNPs. Stability tests conducted over two weeks indicated consistent concentration levels, demonstrating the exceptional stability of the NPs. UV–Vis spectrum analysis supported these findings, showing overlapping peaks over two weeks for both Barley-AuNPs and Barley-AgNPs (Fig. 7E, F). Stability tests in different media and aqueous solutions (Fig. 7G, H), as well as temperature stability analysis (Fig. 7I, J), reinforced the remarkable stability of Barley-AuNPs and Barley-AgNPs [27].

**Table 1:** FT-IR spectra of barley extract, Barley-AuNPs and Barley-AgNPs

Type of bond	Barley extract wavenumber (cm^−1^)	AuNPs wavenumber (cm^−1^)	AgNPs wavenumber (cm^−1^)
O–H strech	3282.08	3277.17	3279.51
C–H strech	2924.74	2928.55	2926.42, 2113.33
C=C strech, COOH strech	1634.09	1637.84, 1534.66	1636.95, 1517.30,
CH_3_, CH_2_, asymmetric deformation	1372.01	1363.55, 1148.47	1365.38, 1148.62, 1076.59
C–O strech	1017.38	1076.13, 996.55, 860.49	999.57, 856.27

**Fig. 8:**
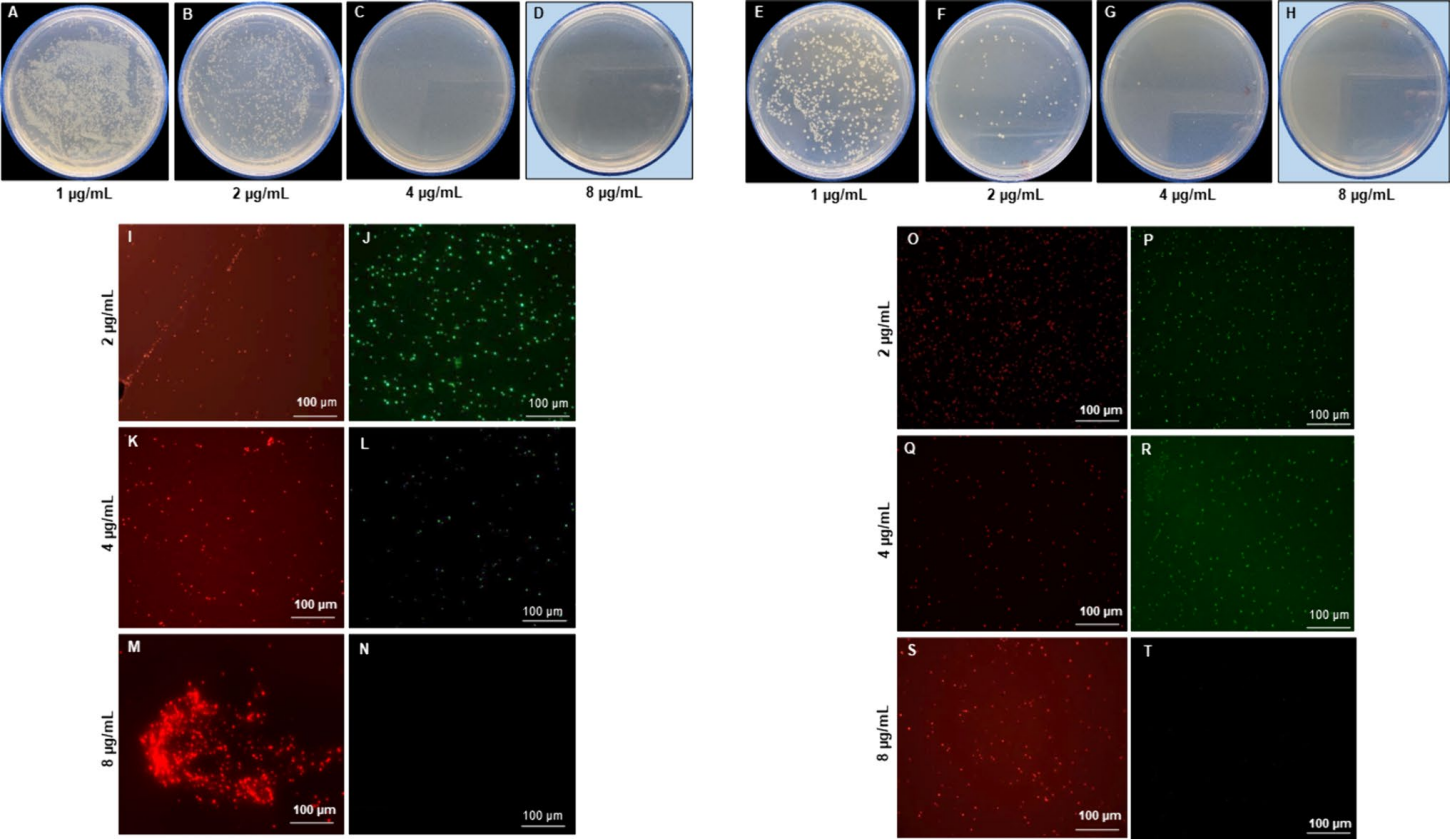
Cell viability test at different concentrations range from 1 to 8 µg/mL of Barley-AgNPs for **A**–**D** *P. aeruginosa* and **E**–**H** *E. coli*. The blue background shows the MBC values of Barley-AgNPs against respective pathogens with complete growth inhibition. Live and dead staining of **I**–**N** *P. aeruginosa* and **O**–**T** *E. coli* cells, after treatment with Barley-AgNPs at different concentrations

**Fig. 9:**
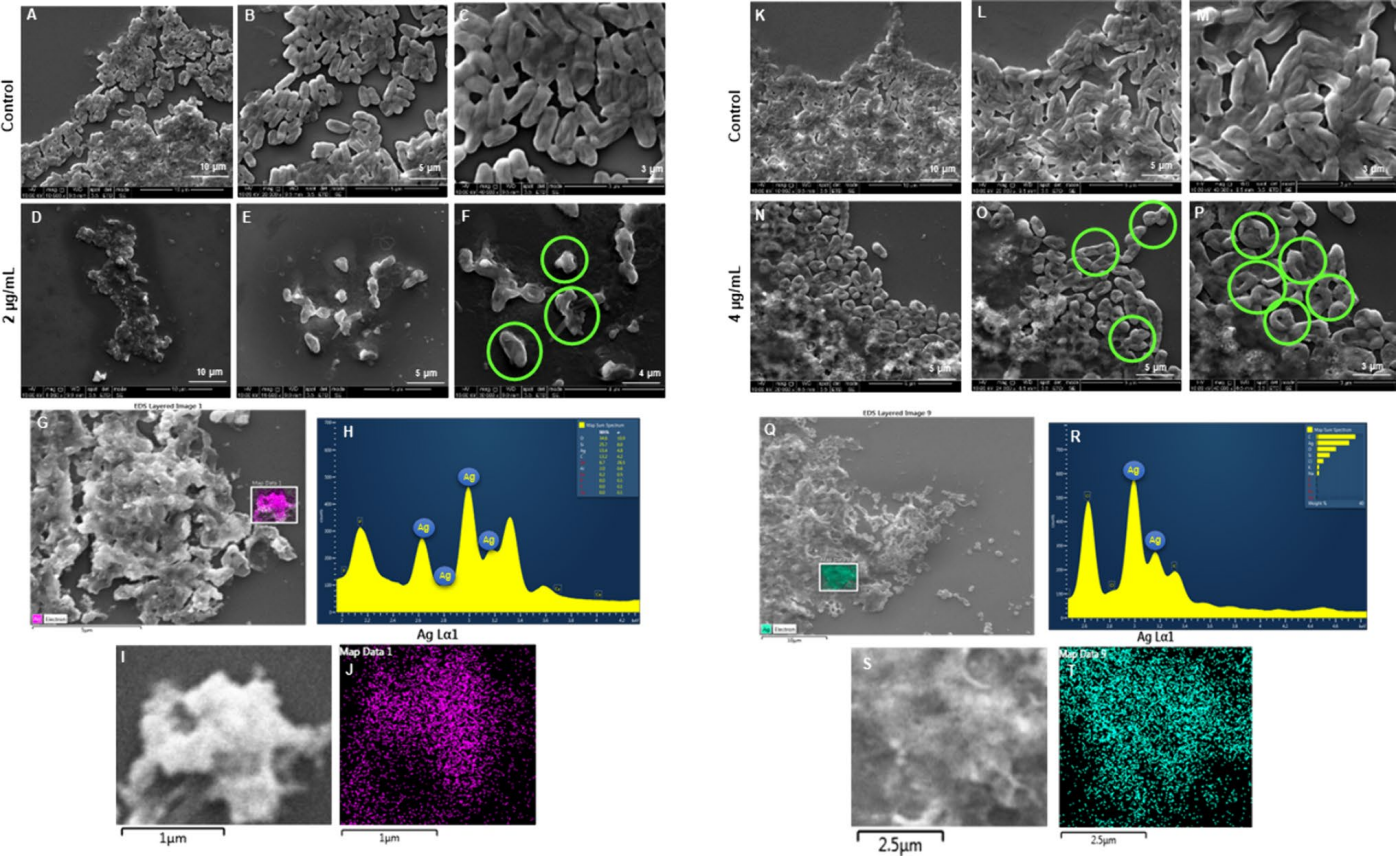
SEM analysis of *P. aeruginosa* cells after treatment with Barley-AgNPs. **A**–**C** Control cells and **D**–**F** Barley-AgNPs treated cells with 2 µg/mL at different scales. **G** Scanned image of treated cells, **H** EDX spectrum of the chosen area showing peak for silver element **I**, **J** elemental mapping of the selected area showing silver element in the treated cells. **K**–**M** Control cells and **N**–**P** Barley-AgNPs treated cells with 4 µg/mL at different scales. **Q** Scanned image of treated cells, **R** EDX spectrum of the chosen area showing peak for silver element **S**, **T** elemental mapping of the selected area showing silver element in the treated cells. Damaged cells are highlighted with green circles

**Fig. 10:**
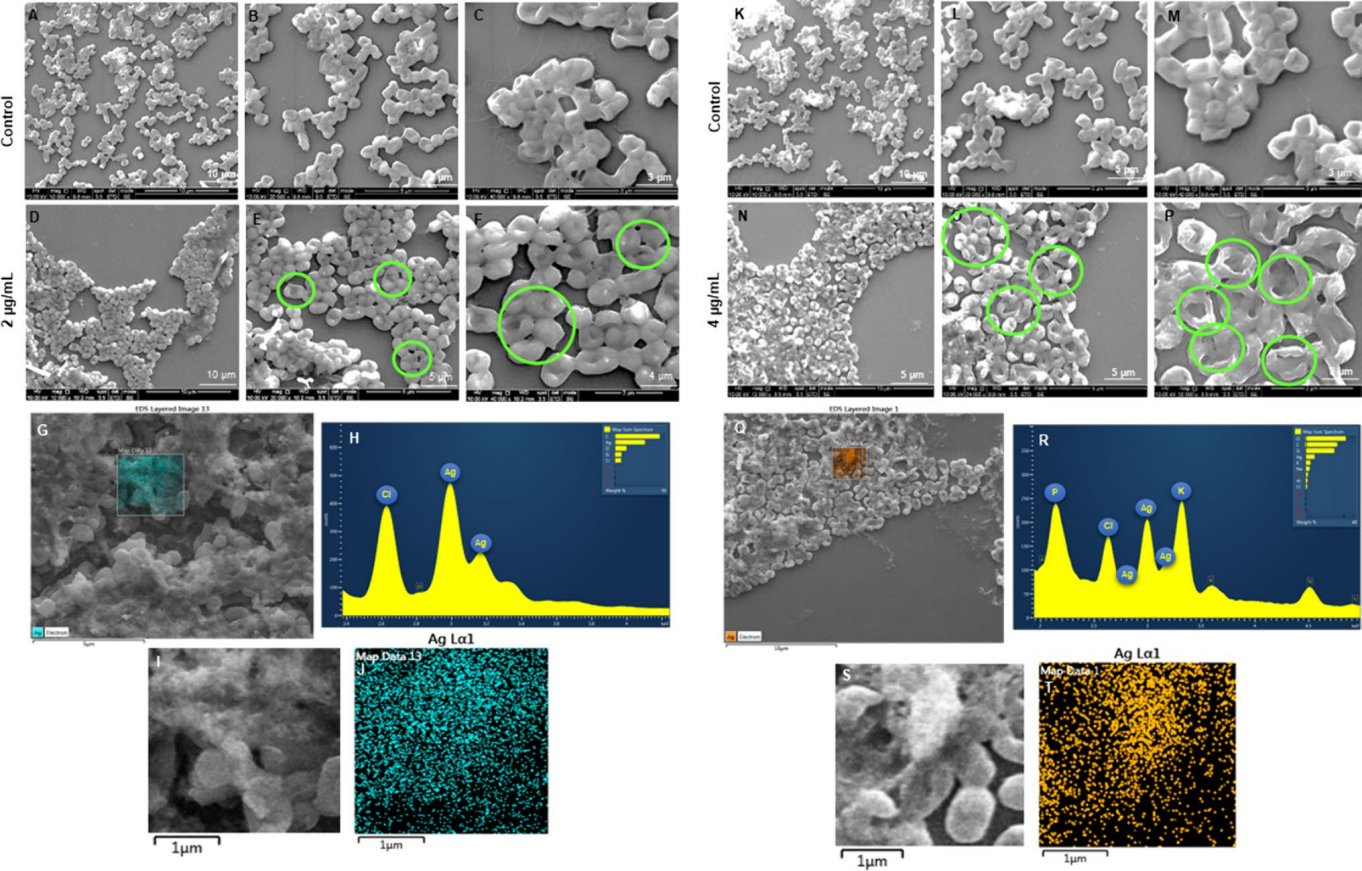
SEM analysis of *E. coli* cells after treatment with Barley-AgNPs. **A**–**C** Control cells and **D**–**F** Barley-AgNPs treated cells with 2 µg/mL at different scales. **G** Scanned image of treated cells, **H** EDX spectrum of the chosen area showing peak for silver element **I**, **J** elemental mapping of the selected area showing silver element in the treated cells. **K**–**M** Control cells and **N**–**P** Barley-AgNPs treated cells with 4 µg/mL at different scales. **Q** Scanned image of treated cells, **R** EDX spectrum of the chosen area showing peak for silver element **S**, **T** elemental mapping of the selected area showing silver element in the treated cells. Damaged cells are highlighted with green circles

The antimicrobial potential of the Barley-AgNPs synthesized in this study was systematically investigated against two Gram-negative bacterial strains, namely *P. aeruginosa* and *E. coli*. Notably, the findings revealed a profound antibacterial effect (Table 2), with AgNPs exhibiting complete eradication of *P. aeruginosa* cells (Fig. 8A-D) and *E. coli* cells at 8 µg/mL, as illustrated in Fig. 8E-H [34]. Live and dead staining techniques were employed to gain further insights into the viability of bacterial cells. Microscopic examination of *P. aeruginosa* cells treated with varying concentrations of Barley-AgNPs demonstrated a transition from predominantly green, viable cells at lower concentrations (8 µg/mL) to a dominance of red, non-viable cells at higher concentrations, affirming the toxic impact of AgNPs on *P. aeruginosa* (Fig. 8I-N). A concentration of 8 µg/mL led to complete cell death [35]. Similarly, for *E. coli* cells (Fig. 8O-T), a concentration of 8 µg/mL resulted in an entire region of red, indicative of extensive cell death. To further characterize the morphological impact of Barley-AgNPs on bacterial cells, SEM was employed. *P. aeruginosa* cells treated with AgNPs at concentrations of 2 and 4 µg/mL exhibited a notable coverage of NPs, leading to open membranes and structural damage (Fig. 9A–F, K-P). Elemental mapping corroborated these observations, indicating comprehensive coverage of damaged cells by AgNPs (Fig. 9G–J, Q-T). The presence of distinct peaks for silver in the EDX analysis further confirmed the purity of AgNPs within the damaged cells. Similar effects were observed for *E. coli* cells treated with Barley-AgNPs, with concentrations of 4 and 8 µg/mL resulting in substantial coverage of cells by silver elements (Fig. 10A–F, K–P). Elemental mapping and EDX analysis provided additional evidence of the antibacterial action of Barley-AgNPs, highlighting the predominant presence of silver within the damaged cells (Fig. 10G–J, Q–T). The findings collectively underscore the antibacterial efficacy of Barley-AgNPs, signifying their potential application in combating Gram-negative bacterial infections [36].

**Table 2:** Minimum inhibitory concentration (MIC) and minimum bactericidal concentration (MBC) of Barley-AgNPs against *P. aeruginosa* and *E. coli*

Bacterial strain	MIC (µg/mL)	MBC (µg/mL)
*P. aeruginosa*	2	8
*E. coli*	2	8

## Discussion

Barley, a semi-evergreen and deciduous shrub found in Europe, North Africa, and Asia, termed *Hordeum vulgare*, has emerged as a valuable resource, readily available in large quantities. By utilizing barley grains for nanoparticle synthesis, this study promotes the valorization of agricultural by-products. Barley grains are commonly used in food, brewing, and animal feed industries, generating significant quantities of waste materials such as spent barley grains. The study contributes to the circular economy concept by repurposing these by-products for nanoparticle synthesis, minimizing waste generation, and maximizing resource efficiency. Here, we explore this abundant plant for the sustainable synthesis of gold and silver NPs. The study demonstrates that this green resource offers an eco-friendly alternative by producing gold and silver NPs at 90 °C within 20–30 min without adding any chemicals to support the reduction or stabilization of formed NPs. This dual functionality simplifies the synthesis process, aligning with principles of simplicity and sustainability by eliminating the need for additional agents. This process circumvents environmental concerns associated with conventional physicochemical methods, which generate hazardous byproducts and consume substantial energy resources [37, 38]. The green synthesis, visibly marked by distinct color changes in the SM, dark purple for Barley-AuNPs, and deep brown for Barley-AgNPs, indicates the successful reduction of gold and silver salts, as corroborated by UV–Vis spectrum analysis [39]. Optimization studies have revealed barley extract's effectiveness in synthesizing NPs as both a reducing and stabilizing agent.

The distinctive morphology of the synthesized NPs was thoroughly investigated using a range of characterization techniques, including SEM, EDX, elemental mapping, TEM, AFM, and DLS. These analyses provided valuable insights into the shape and size distribution of the generated NPs. Particularly noteworthy were the differences observed between the core diameter of NPs as observed with TEM and AFM, compared to the hydrodynamic size measured by DLS. Specifically, 20–30 nm AuNPs appeared to be 1565 nm, and 2–20 nm AgNPs measured up to 4989 nm in hydrodynamic size. Interestingly, the AgNPs exhibited thicker and more complex surrounding layers than the AuNPs. This disparity suggests the presence of a thick corona layer surrounding the NPs, resulting in an apparent increase in size. Importantly, barley grains offer inherent advantages because of their composition, which includes a complex matrix of proteins, carbohydrates, and phytochemicals, which can serve as reducing agents, stabilizers, or capping agents during nanoparticle formation [40]. This multifaceted composition contributes to the stability and biocompatibility of the synthesized nanoparticles, enhancing their suitability for various applications [41]. This discrepancy underscores the importance of considering the corona layer in nanoparticle characterization and its potential influence on nanoparticle properties and behavior, which could also have implications for their antibacterial activity [34, 42].

The significance of the biological corona surrounding NPs has been extensively studied and documented in the literature [42, 43]. This corona layer forms around NPs during synthesis, often originating from the biological components present in the synthesis medium. Beyond merely stabilizing NPs, the corona profoundly influences their properties and activities, including antibacterial efficacy, catalytic performance, and cellular interactions. The composition and structure of the corona are intricately linked to the surrounding biological environment, resulting in dynamic and context-dependent effects on nanoparticle behavior. Understanding the role of the biological corona is crucial for tailoring nanoparticle properties and optimizing their performance in various applications [44, 45]. In the current study, the formed corona is assumed to be composed of biological components from barley, as evidenced by FTIR and MALDI-TOF results, which appear to align with barley extract. The controlled formation of this corona layer demonstrates the intricate interaction between barley components and metal ions during synthesis. The distinctive peaks observed in the FTIR spectra unequivocally indicate the participation of various bioactive compounds in the synthesis of NPs [46]. Notably, the presence of flavanones, evident from characteristic absorption bands, suggests their potential role as reducing or stabilizing agents during the formation of NPs (Table 1). Flavanones, known for their antioxidant properties, may contribute to the overall stability and biocompatibility of the NPs. Proteins and amino acids, integral components of barley, exhibit discernible peaks in the FTIR spectra, pointing towards their active involvement in the synthesis process. These biomolecules can further act as capping agents, facilitating the stabilization of NPs and influencing their size and morphology [47]. The interplay between proteins and metal ions is crucial in governing the nucleation and growth of NPs, contributing to the overall efficacy of the synthesis. Polyphenols, another significant component identified through FT-IR peaks, add another layer of complexity to the synthesis mechanism [31]. Polyphenols, recognized for their antioxidant and antibacterial properties, may impart enhanced functionalities to the NPs, potentially influencing their antimicrobial applications. Moreover, cellulose, a major structural component in barley, manifests its presence through characteristic FT-IR peaks, suggesting its potential role in shaping the nanoparticle morphology. The interaction between cellulose and metal ions during synthesis could impact the overall structure and stability of the NPs. In essence, the FT-IR analysis unveils the diverse bioactive compounds within barley and provides a roadmap for understanding their orchestrated involvement in the synthesis of NPs. The synergistic action of flavanones, proteins, amino acids, polyphenols, and cellulose creates a complex yet finely tuned environment, shaping the physicochemical properties of the resulting NPs [30].

Stability assessments conducted over two weeks, utilizing techniques such as ICPMS and UV–Vis analyses, confirm the enduring stability of Barley-AuNPs and Barley-AgNPs, which correspond to the thick corona layer and its stable nature. Negative zeta potential values further support stability, indicating electrostatic repulsion and steric hindrance as stabilizing forces. Understanding the thermal dynamics is crucial for predicting how barley NPs perform in different environmental conditions [7]. Thermal analysis through TGA provides insights into the NPs' degradability at higher temperatures. The gradual depletion of the corona layer observed in TGA indicates controlled thermal behavior [48]. The combined insights from stability assessments, zeta potential measurements, and other analytical techniques affirm that Barley-AuNPs and Barley-AgNPs possess enduring stability and characteristics conducive to diverse practical applications. These NPs, with stability mechanisms rooted in electrostatic repulsion and steric hindrance, hold promise for sustained performance and adaptability in various conditions [49].

Antibacterial studies with Barley-AgNPs against *P. aeruginosa* and *E. coli* reveal their efficacy, with MBC values of 8 µg/mL, respectively. The plate assay findings indicate a robust bacterial growth observed at a concentration of 2 µg/mL, which undergoes a remarkable reduction upon elevating the concentration to 8 µg/mL for both *P. aeruginosa* and *E. coli* (Fig. 8). This trend is consistently validated by live and dead staining, affirming the loss of viability (depicted in green fluorescence) at the higher concentration of NPs for both bacterial strains. The implications of these observations suggest a concentration-dependent antimicrobial effect of Barley-AgNPs against the studied pathogens. Exploring the structural aspects, SEM unravels noteworthy alterations in the morphology of the treated microbial cells [35]. Specifically, the cells exhibit conspicuous swelling and an open configuration, characteristic of membrane damage. This morphological insight aligns with the proposition that the action of Barley-AgNPs involves perturbation of the bacterial cell membrane. The elemental analysis through EDX and elemental mapping provides further substantiation by revealing a discernible accumulation of silver elements on the damaged regions of the bacterial cells. This spatial correlation underscores the role of AgNPs in inducing structural modifications, supporting the notion that the observed effects are directly attributable to the presence and action of Barley-AgNPs [34]. Thus, live and dead staining experiments, SEM analysis, and elemental mapping confirm the damage to bacterial cells, providing insights into potential mechanisms such as internalization, membrane leakage, and reactive oxygen species (ROS) generation [29]. The multifaceted exploration of these mechanisms provides a nuanced understanding of how barley-derived AgNPs exert their antimicrobial effects. The preferential deposition of silver on compromised cellular regions suggests a targeted mode of action, warranting continued exploration of the molecular mechanisms involved. However, further investigations are imperative to elucidate the specific biochemical pathways involved and to evaluate the translational potential of these NPs in practical antimicrobial interventions. The sustainable synthesis of barley-NPs aligns with the growing demand for eco-friendly and biocompatible nanomaterials [50]. For instance, the thick corona layer observed in barley NPs, while contributing to stability and biocompatibility, might influence antimicrobial efficacy compared to NPs with thinner coatings. Most importantly, the ability of these AgNPs to maintain their appearance after killing pathogenic cells raises intriguing questions about their reusability and environmental impact, warranting further investigation. The multifunctionality of Barley-AgNPs, maintaining stability even after exerting antimicrobial effects, prompts reflections on potential applications beyond antibacterial properties [14, 21].

Overall, the choice of barley plants as a biological source for synthesizing gold and silver nanoparticles leverages their abundant availability and sustainable attributes and underscores their inherent biochemical properties that facilitate efficient and eco-friendly nanoparticle production. Questions regarding the reusability of NPs, considering their interaction with living organisms and potential corona layer alterations, pose intriguing challenges for future research. Here, we didn’t attempt to explore the barley-AuNPs for any antibacterial activity because of the inherent differences in antimicrobial activity between gold and silver nanoparticles. While barely-AgNPs exhibit potent antimicrobial properties, barely-AuNPs generally have lower efficacy, necessitating impractical concentrations for comparable effects. Instead, barley AuNPs could be explored for alternative applications, such as sensors or anticancer therapies, where their unique properties can be effectively leveraged.

## Conclusions

In the face of increasing demand for eco-friendly NPs with enhanced stability and efficacy, our research addressed the challenge of sustainable synthesis by leveraging the abundant and readily available barley species. Through a comprehensive exploration of reaction parameters, we aimed to establish a cost-effective method for producing gold and silver NPs without additional reducing or stabilizing agents. Our findings suggest that utilizing barley extract offers a promising solution to this problem by producing enormous ability and stability NPs. By systematically optimizing the synthesis process, we have successfully demonstrated the efficient production of stable NPs with transparent corona layers, ensuring resilience under ambient conditions. Moreover, the antimicrobial properties of barley-synthesized AgNPs against severe human pathogens *P. aeruginosa* and *E. coli* at 8 µg/mL further highlight the importance of these NPs. Thus, our study effectively addresses the problem of eco-friendly nanoparticle synthesis with higher antibacterial potential by harnessing the potential of barley extract, offering a sustainable pathway toward enhanced stability and efficacy in nanoparticle applications.

## Data Availability

Data is provided within the manuscript.

## References

[1] Rathod S, Preetam S, Pandey C, Bera SP (2024). Exploring synthesis and applications of green nanoparticles and the role of nanotechnology in wastewater treatment. Biotechnol Rep (Amst).

[2] Jiang Y, Zhou P, Zhang P, Adeel M, Shakoor N, Li Y, Li M, Guo M, Zhao W, Lou B, Wang L, Lynch I, Rui Y (2022). Green synthesis of metal-based nanoparticles for sustainable agriculture. Environ Pollut.

[3] Singh P, Pandit S, Garnæs J, Tunjic S, Mokkapati VRSS, Sultan A, Thygesen A, Mackevica A, Mateiu RV, Daugaard AE, Baun A, Mijakovic I (2018). Green synthesis of gold and silver nanoparticles from *Cannabis sativa* (industrial hemp) and their capacity for biofilm inhibition. Int J Nanomed.

[4] Singh P, Mijakovic I (2022). Rowan berries: a potential source for green synthesis of extremely monodisperse gold and silver nanoparticles and their antimicrobial property. Pharmaceutics.

[5] Singh P, Kim Y-J, Zhang D, Yang D-C (2016). Biological synthesis of nanoparticles from plants and microorganisms. Trends Biotechnol.

[6] Bhagat DS, Gurnule WB, Bumbrah GS, Koinkar P, Chawla PA (2023). Recent advances in biomedical applications of biogenic nanomaterials. Curr Pharm Biotechnol.

[7] Hasan KMF, Xiaoyi L, Shaoqin Z, Horváth PG, Bak M, Bejó L, Sipos G, Alpár T (2022). Functional silver nanoparticles synthesis from sustainable point of view: 2000 to 2023: a review on game changing materials. Heliyon.

[8] Nayak S, Goveas LC, Kumar PS, Selvaraj R, Vinayagam R (2022). Plant-mediated gold and silver nanoparticles as detectors of heavy metal contamination. Food Chem Toxicol.

[9] Muddapur UM, Alshehri S, Ghoneim MM, Mahnashi MH, Alshahrani MA, Khan AA, Iqubal SMS, Bahafi A, More SS, Shaikh IA, Mannasaheb BA, Othman N, Maqbul MS, Ahmad MZ (2022). Plant-based synthesis of gold nanoparticles and theranostic applications: a review. Molecules.

[10] Alharbi NS, Alsubhi NS, Felimban AI (2022). Green synthesis of silver nanoparticles using medicinal plants: characterization and application. J Radiat Res Appl Sci.

[11] Balusamy SR, Joshi AS, Perumalsamy H, Mijakovic I, Singh P (2023). Advancing sustainable agriculture: a critical review of smart and eco-friendly nanomaterial applications. J Nanobiotechnol.

[12] Macovei I, Luca SV, Skalicka-Wozniak K, Sacarescu L, Pascariu P, Ghilan A, Doroftei F, Ursu EL, Rimbu CM, Horhogea CE, Lungu C, Vochita G, Panainte AD, Nechita C, Corciova MA, Miron A (2021). Phyto-functionalized silver nanoparticles derived from conifer bark extracts and evaluation of their antimicrobial and cytogenotoxic effects. Molecules.

[13] Singh P, Mijakovic I (2022). Green synthesis and antibacterial applications of gold and silver nanoparticles from *Ligustrum vulgare* berries. Sci Rep.

[14] Miranda RR, Sampaio I, Zucolotto V (2022). Exploring silver nanoparticles for cancer therapy and diagnosis. Colloids Surf B.

[15] Fan M, Han Y, Gao S, Yan H, Cao L, Li Z, Liang XJ, Zhang J (2020). Ultrasmall gold nanoparticles in cancer diagnosis and therapy. Theranostics.

[16] Raiche-Marcoux G, Loiseau A, Maranda C, Poliquin A, Boisselier E (2022). Parametric drug release optimization of anti-inflammatory drugs by gold nanoparticles for topically applied ocular therapy. Int J Mol Sci.

[17] Li B, Lane LA (2019). Probing the biological obstacles of nanomedicine with gold nanoparticles. Wiley Interdiscip Rev Nanomed Nanobiotechnol.

[18] Amina SJ, Guo B (2020). A review on the synthesis and functionalization of gold nanoparticles as a drug delivery vehicle. Int J Nanomed.

[19] Bruna T, Maldonado-Bravo F, Jara P, Caro N (2021). Silver nanoparticles and their antibacterial applications. Int J Mol Sci.

[20] Haji SH, Ali FA, Aka STH (2022). Synergistic antibacterial activity of silver nanoparticles biosynthesized by carbapenem-resistant gram-negative bacilli. Sci Rep.

[21] Ronavari A, Igaz N, Adamecz DI, Szerencses B, Molnar C, Konya Z, Pfeiffer I, Kiricsi M (2021). Green silver and gold nanoparticles: biological synthesis approaches and potentials for biomedical applications. Molecules.

[22] Joshi AS, Singh P, Mijakovic I (2020). Interactions of gold and silver nanoparticles with bacterial biofilms: molecular interactions behind inhibition and resistance. Int J Mol Sci.

[23] da Costa TS, da Silva MR, Jeronimo Barbosa JC, Da Silva Das Neves U, de Jesus MB, Tasic L (2024). Biogenic silver nanoparticles' antibacterial activity and cytotoxicity on human hepatocarcinoma cells (Huh-7). RSC Adv.

[24] Singh P, Garg A, Pandit S, Mokkapati VRSS, Mijakovic I (2018). Antimicrobial effects of biogenic nanoparticles. Nanomaterials.

[25] Singh P, Pandit S, Mokkapati V, Garnæs J, Mijakovic I (2020). A sustainable approach for the green synthesis of silver nanoparticles from *Solibacillus isronensis* sp. and their application in biofilm inhibition. Molecules.

[26] Singh P, Pandit S, Jers C, Joshi AS, Garnæs J, Mijakovic I (2021). Silver nanoparticles produced from Cedecea sp. exhibit antibiofilm activity and remarkable stability. Sci Rep.

[27] Singh P, Mijakovic I (2022). Antibacterial effect of silver nanoparticles is stronger if the production host and the targeted pathogen are closely related. Biomedicines.

[28] Singh P, Pandit S, Beshay M, Mokkapati V, Garnaes J, Olsson ME, Sultan A, Mackevica A, Mateiu RV, Lutken H, Daugaard AE, Baun A, Mijakovic I (2018). Anti-biofilm effects of gold and silver nanoparticles synthesized by the *Rhodiola rosea* rhizome extracts. Artif Cells Nanomed Biotechnol.

[29] Singh P, Mijakovic I (2022). Strong antimicrobial activity of silver nanoparticles obtained by the green synthesis in *Viridibacillus* sp. Extr Front Microbiol.

[30] Soliman MKY, Salem SS, Abu-Elghait M, Azab MS (2023). Biosynthesis of silver and gold nanoparticles and their efficacy towards antibacterial, antibiofilm, cytotoxicity, and antioxidant activities. Appl Biochem Biotechnol.

[31] Veeragoni D, Deshpande SS, Singh V, Misra S, Mutheneni SR (2023). In vitro and in vivo antimalarial activity of green synthesized silver nanoparticles using *Sargassum tenerrimum*—a marine seaweed. Acta Trop.

[32] Sandulovici RC, Carmen-Marinela M, Grigoroiu A, Moldovan CA, Savin M, Ordeanu V, Voicu SN, Cord D, Costache GM, Galatanu ML, Popescu M, Sarbu I, Mati E, Ionescu LE, Neagu R, Tucureanu V, Claudia RM, Mihalache I, Romanitan C, Piperea-Sianu A, Boldeiu A, Brincoveanu O, Manea CE, Firtat B, Muscalu GS, Dragomir D (2022). The physicochemical and antimicrobial properties of silver/gold nanoparticles obtained by “green synthesis” from willow bark and their formulations as potential innovative pharmaceutical substances. Pharmaceuticals (Basel).

[33] Urnukhsaikhan E, Bold B-E, Gunbileg A, Sukhbaatar N, Mishig-Ochir T (2021). Antibacterial activity and characteristics of silver nanoparticles biosynthesized from *Carduus crispus*. Sci Rep.

[34] Liu X, Chen J-L, Yang W-Y, Qian Y-C, Pan J-Y, Zhu C-N, Liu L, Ou W-B, Zhao H-X, Zhang D-P (2021). Biosynthesis of silver nanoparticles with antimicrobial and anticancer properties using two novel yeasts. Sci Rep.

[35] Sikdar S, Sikdar M (2023). Green synthesis, optimization and analyzing of silver nanoparticles encapsulated with *Syzygium aromaticum* extract: evaluating antibacterial and photocatalytic properties. Bioresour Technol Rep.

[36] Ajlouni A-W, Hamdan EH, Alshalawi RAE, Shaik MR, Khan M, Kuniyil M, Alwarthan A, Ansari MA, Khan M, Alkhathlan HZ, Shaik JP, Adil SF (2023). Green synthesis of silver nanoparticles using aerial part extract of the A*nthemis pseudocotula Boiss*. Plant Their Biol Activity Mol.

[37] Aswathi VP, Meera S, Maria CGA, Nidhin M (2023). Green synthesis of nanoparticles from biodegradable waste extracts and their applications: a critical review. Nanotechnol Environ Eng.

[38] Shanmugam J, Dhayalan M, Savaas Umar MR, Gopal M, Ali-Khan M, Simal-Gandara J, Cid-Samamed A (2022). Green synthesis of silver nanoparticles using *Allium cepa* var. Aggregatum natural extract: antibacterial and cytotoxic properties. Nanomaterials (Basel).

[39] Do-Dat T, Cong CQ, Le-Hoai-Nhi T, Khang PT, Nam NTH, Thi-Tinh N, Hue DT, Hieu NH (2023). Green synthesis of gold nanoparticles using *Andrographis paniculata* leave extract for lead ion detection, degradation of dyes, and bioactivities. Biochem Eng J.

[40] Chugh G, Singh BR, Adholeya A, Barrow CJ (2022). Role of proteins in the biosynthesis and functioning of metallic nanoparticles. Crit Rev Biotechnol.

[41] Park Y, Hong YN, Weyers A, Kim YS, Linhardt RJ (2011). Polysaccharides and phytochemicals: a natural reservoir for the green synthesis of gold and silver nanoparticles. IET Nanobiotechnol.

[42] Li Y, Lee JS (2020). Insights into characterization methods and biomedical applications of nanoparticle-protein corona. Materials (Basel).

[43] Gonzalez-Garcia LE, MacGregor MN, Visalakshan RM, Lazarian A, Cavallaro AA, Morsbach S, Mierczynska-Vasilev A, Mailander V, Landfester K, Vasilev K (2022). Nanoparticles surface chemistry influence on protein corona composition and inflammatory responses. Nanomaterials (Basel).

[44] Shourni S, Javadi A, Hosseinpour N, Bahramian A, Raoufi M (2022). Characterization of protein corona formation on nanoparticles via the analysis of dynamic interfacial properties: bovine serum albumin—silica particle interaction. Colloids Surf A.

[45] Breznica P, Koliqi R, Daka A (2020). A review of the current understanding of nanoparticles protein corona composition. Med Pharm Rep.

[46] Khan F, Jeong GJ, Singh P, Tabassum N, Mijakovic I, Kim YM (2022). Retrospective analysis of the key molecules involved in the green synthesis of nanoparticles. Nanoscale.

[47] Saeed Z, Pervaiz M, Ejaz A, Hussain S, Shaheen S, Shehzad B, Younas U (2023). Garlic and ginger extracts mediated green synthesis of silver and gold nanoparticles: a review on recent advancements and prospective applications’. Biocatal Agric Biotechnol.

[48] Abdelsattar AS, Kamel AG, Hussein AH, Azzam M, Makky S, Rezk N, Essam K, Agwa MM, El-Shibiny A (2023). The promising antibacterial and anticancer activity of green synthesized zinc nanoparticles in combination with silver and gold nanoparticles. J Inorg Organomet Polym Mater.

[49] Keskin C, Olcekci A, Baran A, Baran MF, Eftekhari A, Omarova S, Khalilov R, Aliyev E, Sufianov A, Beilerli A, Gareev I (2023). Green synthesis of silver nanoparticles mediated *Diospyros kaki* L. (Persimmon): determination of chemical composition and evaluation of their antimicrobials and anticancer activities. Front Chem.

[50] Viswanathan S, Palaniyandi T, Shanmugam R, Karunakaran S, Pandi M, Wahab MRA, Baskar G, Rajendran BK, Sivaji A, Moovendhan M (2023). Synthesis, characterization, cytotoxicity, and antimicrobial studies of green synthesized silver nanoparticles using red seaweed *Champia parvula*. Biomass Convers Biorefinery.

